# Use of Indirect Target Gating in Magnetic Resonance-guided Liver Stereotactic Body Radiotherapy: Case Report of an Oligometastatic Patient

**DOI:** 10.7759/cureus.2292

**Published:** 2018-03-09

**Authors:** Luca Boldrini, Francesco Cellini, Stefania Manfrida, Giuditta Chiloiro, Stefania Teodoli, Davide Cusumano, Bruno Fionda, Gian Carlo Mattiucci, Anna Maria De Gaetano, Luigi Azario, Vincenzo Valentini

**Affiliations:** 1 Radiology, Polo Scienze Oncologiche Ed Ematologiche, Istituto Di Radiologia, Università Cattolica Del Sacro Cuore, Fondazione Policlinico Universitario Agostino Gemelli, Rome, Italy; 2 Polo Scienze Oncologiche Ed Ematologiche, Università Cattolica Del Sacro Cuore - Fondazione Policlinico Universitario Agostino Gemelli, Roma - Italy; 3 Polo Scienze Oncologiche Ed Ematologiche, Istituto Di Radiologia, Università Cattolica Del Sacro Cuore - Fondazione Policlinico Universitario Agostino Gemelli, Roma - Italy; 4 Medical Physics, Polo Scienze Oncologiche Ed Ematologiche, Istituto Di Fisica, Università Cattolica Del Sacro Cuore - Fondazione Policlinico Universitario Agostino Gemelli, Roma - Italy; 5 Radiology, Polo Scienze Oncologiche Ed Ematologiche, Istituto Di Radiologia, Università Cattolica Del Sacro Cuore - Fondazione Policlinico Universitario Agostino Gemelli, Roma - Italy; 6 Radiology, Polo Scienze Delle Immagini, Di Laboratorio E Infettivologiche, Istituto Di Radiologia, Università Cattolica Del Sacro Cuore - Fondazione Policlinico Universitario Agostino Gemelli, Roma - Italy

**Keywords:** mr guided radiotherapy, gating, igrt, sbrt, oligometastatic, liver sbrt

## Abstract

The case of a 73-year-old woman affected by anal canal cancer with concomitant liver metastases is presented here. The patient was addressed to stereotactic body radiotherapy (SBRT) on two hepatic secondary lesions after the first radiochemotherapy treatment of the primary tumor. A Tri-60-Co magnetic resonance hybrid radiotherapy unit was used for SBRT treatment delivery. Both liver lesions were not clearly visible on the setup magnetic resonance imaging (MRI) due to their limited dimensions (maximum diameter 13 mm); however, the presence of two cysts adjacent to the metastases allowed the use of an indirect target gating approach. Treatment was delivered in deep inspiration breath-hold conditions using the visual feedback technique for breathing control optimization. Post radiotherapy imaging assessed the complete response.

## Introduction

Cancer patients presenting a limited number of organ-confined metastases have recently been defined as “oligometastatic” and because of the more aggressive multidisciplinary oncological treatments that target the secondary lesions, long-term survival is often achieved. Hepatic secondary lesions represent the most common disease presentation in this category of patients and their local treatment (such as surgery, cryoablation, radiofrequency or microwave ablation, and radiotherapy) is associated with encouraging prognostic outcomes. In particular, stereotactic body radiation therapy (SBRT) is capable of reaching local control rates as high as 100% at one year and represents a safe and appealing alternative to surgery. Nevertheless, it has to be considered that up to 90% of liver metastases results are unresectable, due to either disease features or the patient’s clinical conditions [1-2].

## Case presentation

History

A 73-year-old lady with severe anal pain and rectal bleeding was submitted to colonoscopy in March 2017. She previously suffered from glaucoma and depression. An outgrowing neoplasm of the anal canal was detected 3 cm from the anal margin; the pathologic report disclosed a squamous carcinoma. At the first oncologic assessment, the patient's general conditions were considered stable and the performance status was scored as Eastern Cooperative Oncology Group (ECOG) 1 for depression, local pain, and rectal bleeding.

Staging radiological findings

**Figure 1:**
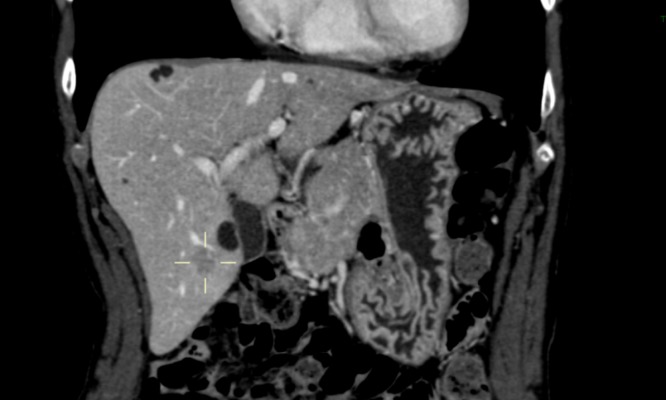
First diagnostic CECT imaging (April 2017) Cystic lesions (with arrows) and V segment metastasis (grey arrow). contrast-enhanced computed tomography: CECT

The staging CT imaging acquired in April 2017 confirmed the presence of a known neoplasm, which measured 5.5 cm in the axial diameter and 8 cm in the cranial-caudal extension. The primary lesion infiltrated the presacral space, the left piriformis muscle, and the perirectal space. The rectovaginal septum and vagina were involved too, and several perirectal nodal lesions were described as radiologically suspect or clearly positive. No secondary lesions were detected in the lungs, while three liver metastases were observed: one in segment VIII (maximum diameter 31 mm) and two in segment V (maximum diameters 13 and 5 mm, respectively). According to the radiological findings, the disease was staged as cT4 cN1 cM1 (liver), stage IV. The case was discussed in the institutional multidisciplinary tumor board for lower gastrointestinal malignancies, and it was decided not to perform further radiological investigations, promptly referring the patient to a protection colostomy and concurrent, long-course chemoradiotherapy. See Figure 1 for computed tomography (CT) hepatic findings.

Chemoradiotherapy on the primary site

Colostomy was performed in May 2017 and chemoradiotherapy was started in June 2017 with the following schedule: 55 Gy in 2.2 Gy fractions to the primary lesion with infiltrated surrounding tissue, infiltrated presacral space, left piriformis muscle, rectovaginal septum, and vagina (CTV1); 55 Gy in 2 Gy fractions to the positive per-rectal nodal lesions (CTV2); 45 Gy in 1.8 Gy fractions to the pelvic, mesorectal, and inguinal nodes (CTV3). The radiation dose has been delivered through the simultaneous integrated boost (SIB) protocol. Concomitant chemotherapy with 5-fluorouracil (1000 mg/m^2^ on days 1-4 and 22-25) and mitomycin C (10 mg/m^2^ on days 1 and 22) was administered during the first and last weeks of radiotherapy. The aforementioned treatment was performed using the volumetric arc therapy (VMAT) delivery technique (RapidArc, Varian, Palo Alto, CA, USA) on a standard X10 MV linear particle accelerator (Linac) and ended on July 18, 2017. The first clinical revaluation was performed 10 days after treatment; the patient suffered from severe tenesmus, mucorrhea, and moderate fatigue. Her ECOG PS score was 2.

Restaging radiological findings after chemoradiotherapy on the primary site

**Figure 2:**
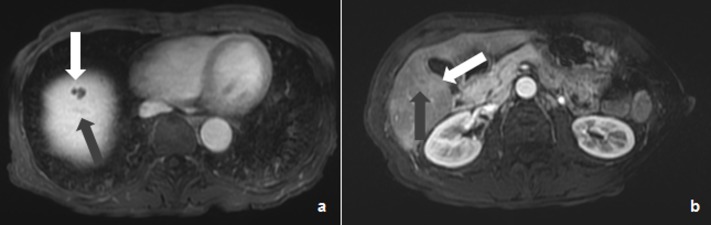
Post-chemotherapy LAVA MR imaging (August 2017) Cystic lesion (white arrow) and residual VIII segment lesion (grey arrow) (a) and cystic lesion (white arrow) and residual V segment lesion (grey arrow) (b). MR: magnetic resonance

A restaging CT of the thorax and an abdominal MRI were performed in August 2017. The CT scan confirmed the absence of secondary lesions in the lungs. The abdominal and pelvic MRIs showed a significant reduction in the primary lesion (the cranial-caudal extension was reduced from 8 cm to 2.5 cm); neoplastic infiltration of the left pararectal space, left piriformis muscle, and rectovaginal septum was still present as a consequence of a limited local response to chemoradiotherapy. The secondary lesion described in the VIII hepatic segment showed a significant dimensional reduction (15x7 mm versus 31x18 mm), while only one lesion was still detectable in segment V (9 mm versus 13 mm). See Figure 2.

Magnetic Resonance-guided Radiotherapy (MRgRT) SBRT for hepatic lesions

Considering the partial response on both the primary and secondary lesions, it was decided to refer the patient to SBRT treatment for the residual hepatic lesions. In consideration of the patient’s general condition and PS (ECOG 2), a total dose of 3500 cGy in five fractions (to be delivered every other day) was prescribed on both lesions. A significant respiratory movement was expected in relation to their location (V and VIII hepatic segments) and, therefore, the patient was referred to MR-guided respiratory-gated tri-60-Cobalt radiotherapy (MRIdian, ViewRay Inc., Mountain View, California, USA) [3]. Specific informed consent for MRgRT was acquired from the patient during a dedicated, extensive clinical interview during which the procedures were described in full detail in order to ensure the patient’s active collaboration during SBRT, as well as the necessary training for the use of the visual feedback system. Complete blood count and hepatic function panel were performed before radiotherapy. The patient received specific training on breath control, free breathing (FB), deep inspiration breath-hold (DIBH), and deep expiration breath-hold (DEBH). A simulation MRI was acquired with 175, 25, and 25 seconds (s) sequences, respectively. CT simulation imaging was then acquired according to the breathing phase on a helical CT scanner (GE HiSpeed DX/i Spiral, Boston, MA, USA); the slice thickness was 3 mm and no intravenous contrast agent was administered, following the simulation protocol of our institution. The patient was immobilized in the supine position with both arms up, using the Fluxboard device (FluxBoard, MacroMedics, The Netherlands) in an appropriate and comfortable configuration (U-grip 11, elbow position Q, black leg support in position 73, feet fix 29). DIBH MR and CT were chosen as the planning imaging, as the patient showed an acceptable attitude to maintain this condition.

Therapy volumes contouring

**Figure 3:**
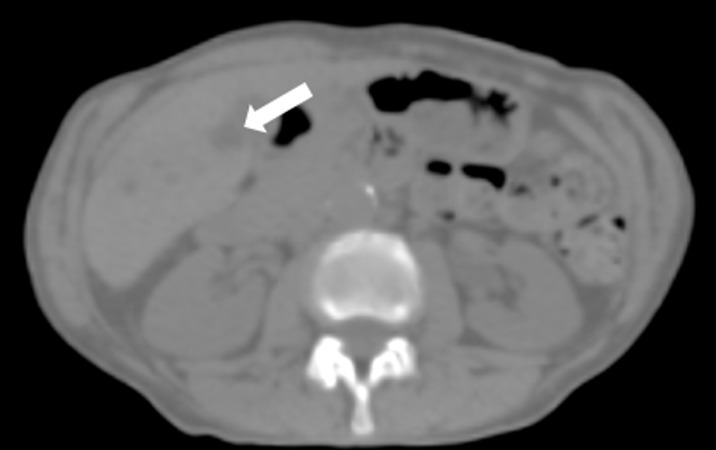
V segment cystic lesion is barely visible Only the V segment cystic lesion is barely visible (white arrow).

The restaging diagnostic CT and MR imaging were fused with the simulation MR and utilized for target identification. Lesions were not visible on simulation CT imaging (see Figure 3).

The identification of target volumes proved to be challenging on simulation MRI too, due to the good response to chemotherapy. A radiologist expert in liver lesion diagnosis was involved in target contouring and margin definition. An anisotropic margin of 0.5 cm was added to the V hepatic segment lesion’s gross tumor volume (GTV) (2.8 cc) in the cranial-caudal direction and of 0.2 cm in the lateral one, obtaining the PTV V segment (12.5 cc). An isotropic margin of 0.6 cm was added to the VIII hepatic segment lesion’s GTV (1.8 cc) in all directions, except for the left side facing the adjacent cystic lesion, obtaining the planning target volume (PTV) VIII segment (7.2 cc). Two cystic lesions were identified very close to both PTV volumes and contoured as cyst V (3.0 cc) and cyst VIII (1.5 cc). The upper abdominal organs at risk (OaR) were contoured according to the usual internal protocols: bowel loops, right and left kidneys, aorta, inferior cava vein, lung, and healthy liver were considered.

MRgRT planning and delivery

Both target volumes (PTV V segment and VIII segment) were not clearly visible on the cine MR during the simulation imaging acquisition: an indirect target gating protocol was therefore adopted, using cyst V and cyst VIII as the target gating volumes. An isotropic boundary of 0.3 cm was set for both volumes.

A. Lesion of the V hepatic segment: Treatment parameters were set as ROI 5%; delay time: 0 s; and wait time: 120 s. Visual feedback for gating purposes was used: the beam on time resulted to be 4.12 minutes, while the average irradiation time was 21 minutes per fraction.

B. Lesion of the VIII hepatic segment: Treatment parameters were set as ROI 5%; delay time: 0 s; and wait time: 120 s. Visual feedback for gating purposes was used: beam on time resulted to be nine minutes, while the average irradiation time was 14 minutes per fraction.

**Figure 4:**
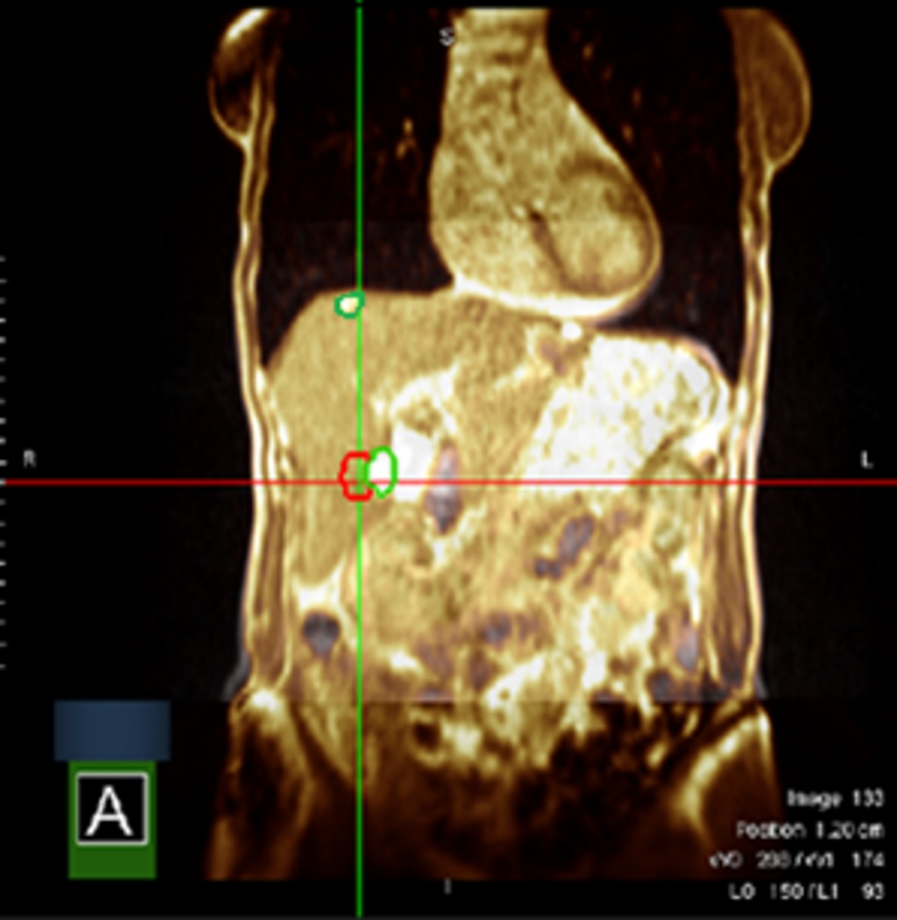
Simulation MR segmentation Cystic lesions of segments V and VIII in green, segment VIII target lesion in red. Segment V target lesion is not visible on the selected plane. Magnetic resonance: MR

Two different plans were calculated in the same DIBH conditions for sequential delivery, one for each lesion. See Figure 4.

Restaging after hepatic SBRT and tolerance assessment

**Figure 5:**
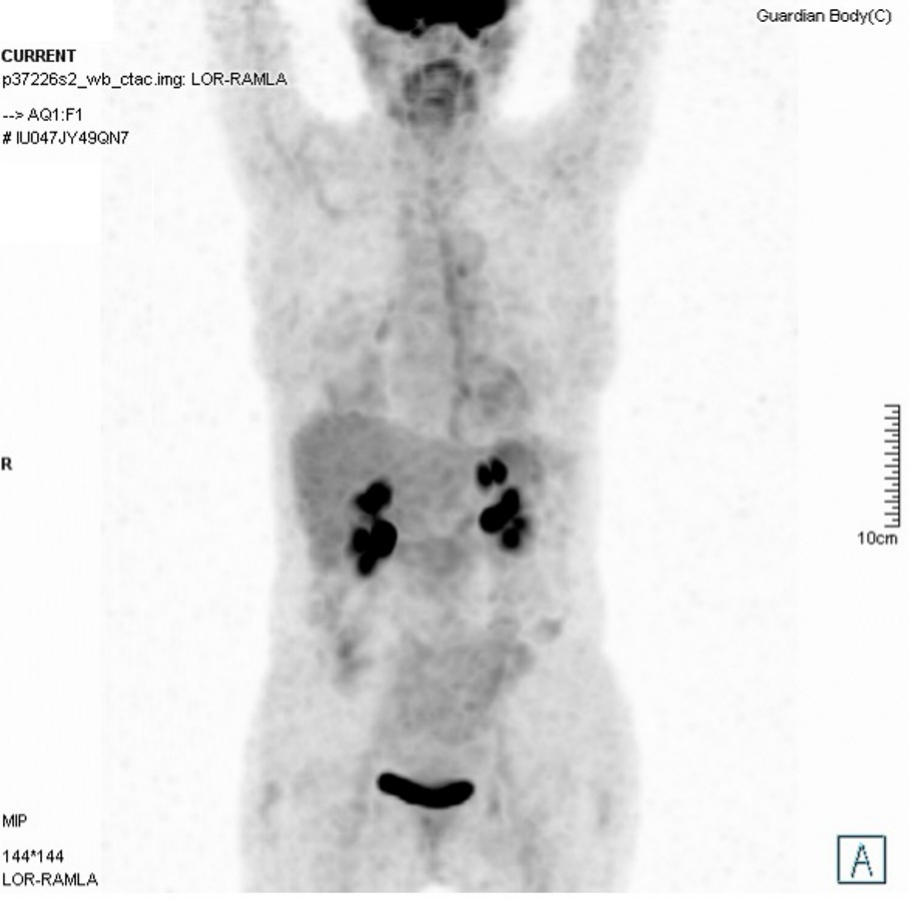
Restaging 18 FDG PET-CT imaging (February 2018) Restaging 18 FDG PET-CT imaging (February 2018) shows a complete response to the treatment. Fluorodeoxyglucose: FDG; positron emission tomography: PET

The patient showed optimal treatment compliance. The result of the complete blood count and hepatic function panel was always in range during and after treatment. Restaging imaging showed a complete response in the irradiated lesions. See Figure 5.

## Discussion

The hybrid MRI-guided radiotherapy technology provides high-quality positioning and gating imaging with a good morphological definition of the soft tissues, especially when compared to the usual onboard kilovoltage (KV) cone-beam computed tomography (CBCT) that produces only a rough visualization of the therapy volumes or their relative surrogates. Furthermore, these therapy imaging developments reduce delineation uncertainties, enhancing the visualization of targets and critical structures and could allow a significant reduction in target margins with immediate effects on the treatment plan quality, both in terms of toxicity decrease and target coverage with the possibility of previously not achievable levels of dose escalation that can potentially avoid radical surgery approaches and preserve the patients’ quality of life. Various strategies have been explored in SBRT literature to take into account radiotherapy volumes motion, such as breathing control techniques and gating or tracking solutions. All these techniques are generally based on the use of radiopaque implants for therapy volume positioning confirmation and delivery monitoring. The recently available real-time cine planar MRI appears able to successfully monitor different types of treatments (i.e. SBRT or intensity-modulated radiation therapy (IMRT)) in various anatomic sites and in all the different breathing phases (free breathing or breath-hold), providing reliable gating structures for such dynamic treatments and overcoming the need to implant the aforementioned invasive markers, limiting patient discomfort (especially in patients considered clinically frail), additional clinical risks, and medical costs. These technological advancements allow, therefore, the safe delivery of ablative doses in the most convenient target volumes and organs at risk position.

## Conclusions

The presented experience suggests that the indirect target-gating approach is feasible and effective in SBRT treatments for liver lesions, even if not clearly visible on MR setup imaging. The use of visual feedback techniques for respiratory motion management enhances treatment quality, reducing the single fraction’s time and improving delivery reliability.
